# Supplementary material for: Astral hydrogels mimic tissue mechanics by aster-aster interpenetration
Source: Nat Commun. 2021 Jul 13;12:4277. doi: 10.1038/s41467-021-24663-y (PMC8277779; doi:10.1038/s41467-021-24663-y)
Supplement: Supplementary file 1 — Supplementary information [file 41467_2021_24663_MOESM1_ESM.pdf]

**Supplementary Information for**

# Astral hydrogels mimic tissue mechanics by aster-aster interpenetration

Qingqiao Xie, Yuandi Zhuang, Gaojun Ye, Tiankuo Wang, Yi Cao, and Lingxiang Jiang*

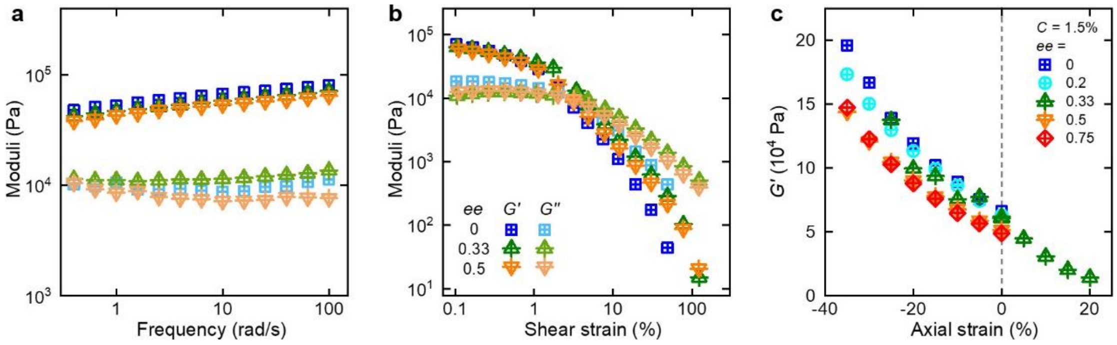


**Supplementary Figure 1.** In frequency sweep (**a**) and shear strain sweep (**b**) experiments, samples with *ee* = 0 or 0.5 behavior similarly to the *ee* = 0.33 sample (concentration = 1.5 wt %). Panel **c** is a copy of Fig. 3e, showing that the slope of the compression-stiffening response is largely invariant to *ee*. In the main text, we reasoned that the compression-stiffening is mostly affected by the overall astral geometry (for example, ribbon density) rather than any molecular specifics or ribbon properties (such as persistence length or helicity). Therefore, the compression-stiffening slope is invariant to *ee* as we observed.

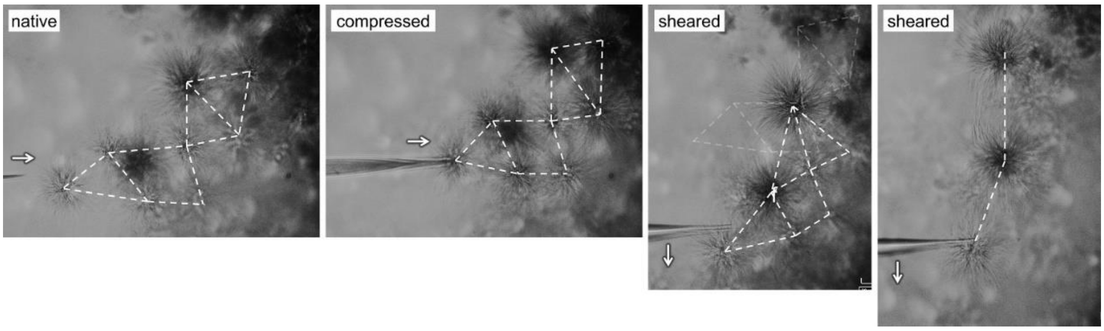


**Supplementary Figure 2.** Deformation of a cluster of asters (*ee* = 0.33, concentration = 1.5 wt%) by an optical fiber. The dashed lines highlight the distance between aster centers.

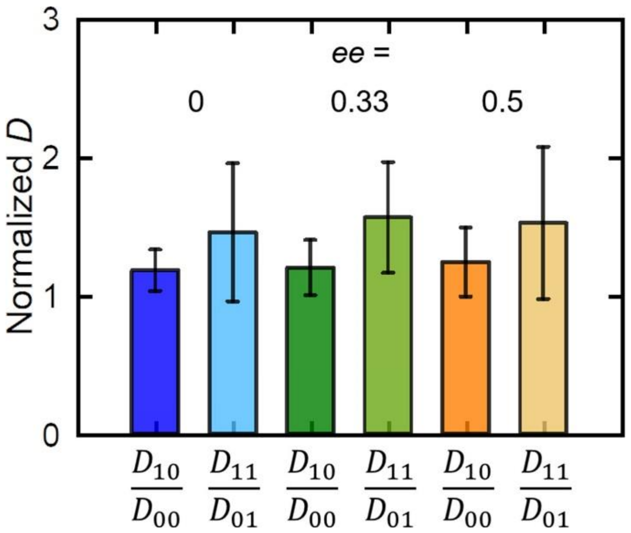


**Supplementary Figure 3.** $D_{10}/D_{00}$ and $D_{11}/D_{10}$ reflects microscopic yield points in uncompressed and pre-compressed states, respectively. Notably, $D_{11}/D_{10}$ is larger than $D_{10}/D_{00}$ in a similar fashion for ee = 0, 0.33, and 0.5. This result suggests that the compression-stiffening response is microscopically similar for samples with different ee values. Error bars represent Standard Deviations (n = 4-5).

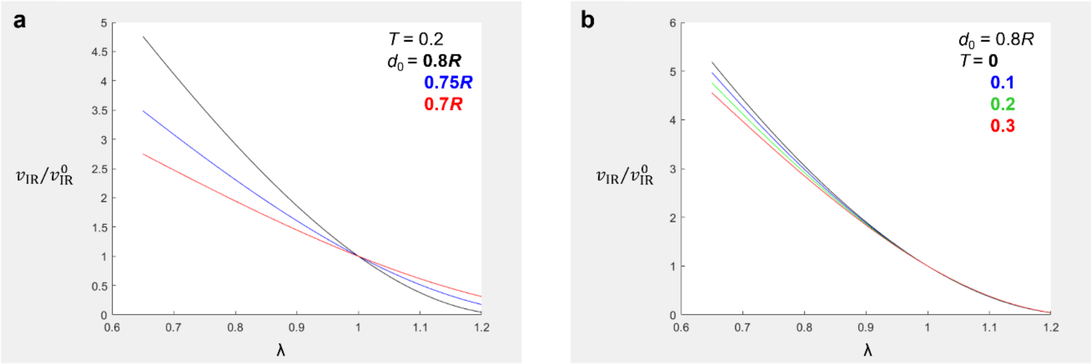


**Supplementary Figure 4.** Volume of interpenetration region as a function of stretch ratio. Effects of input parameters ($d_0$ and $T$) are demonstrated.

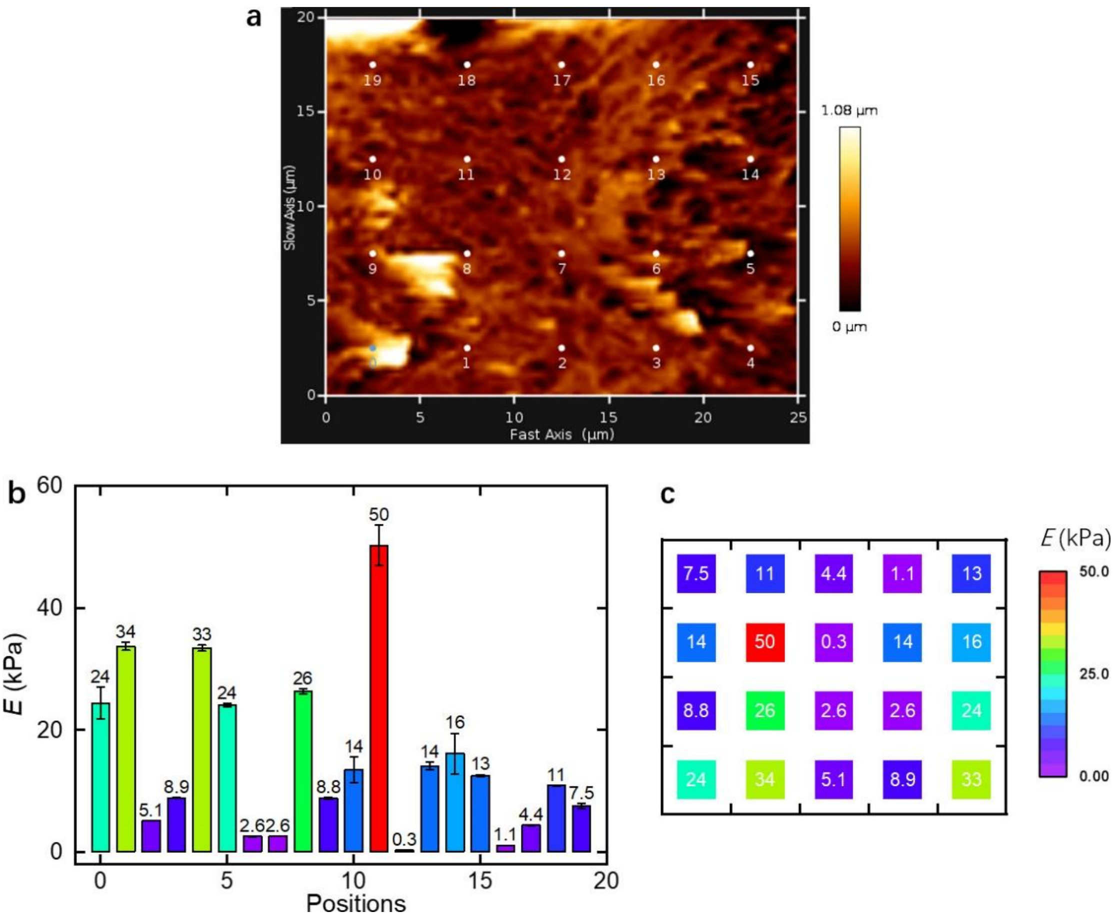


**Supplementary Figure 5. a**, Gel surface height fluctuates on the order of 500 nm as measured by AFM, where regularly separated positions on a lattice are labeled as 0 to 19. Color bar indicates height in z direction. Young's moduli ($E$) of the above positions are summarized in **b** and **c**, where they share a common color bar to represent $E$ from 0 to 50 kPa. Error bars in **b** represent standard deviations for repeated measurements (n = 200-300).

Notably, the spatial variation is significantly large from 0.3 to 50 kPa, suggesting that inhomogeneity in aster sizes and packing could affect local mechanical properties. There is no obvious spatial correlation in modulus in panel c. The overall mean $E$ is 15 kPa, in agreement with $G'$ = 6 kPa measured by rheometer ($E = 3G'$ assuming a Poisson's ratio of 0.5).

We would like to add a few words of explanation on the selection of spatially resolved mechanics methods. While AFM and microrheology techniques are routinely used to probe local mechanics, they are, as far as we know, still limited to the uniaxial responses. It is rather difficult to locally measure biaxial mechanics (e.g., compression

in the vertical direction and stiffening in the horizontal direction) by both methods. For example, the AFM cantilever is perfectly suited to vertically deform the asters, but it is hard to shear the asters horizontally. Therefore, the spatial variation in Young's modulus as we measured in this section cannot imply the variation of compression-stiffening response.

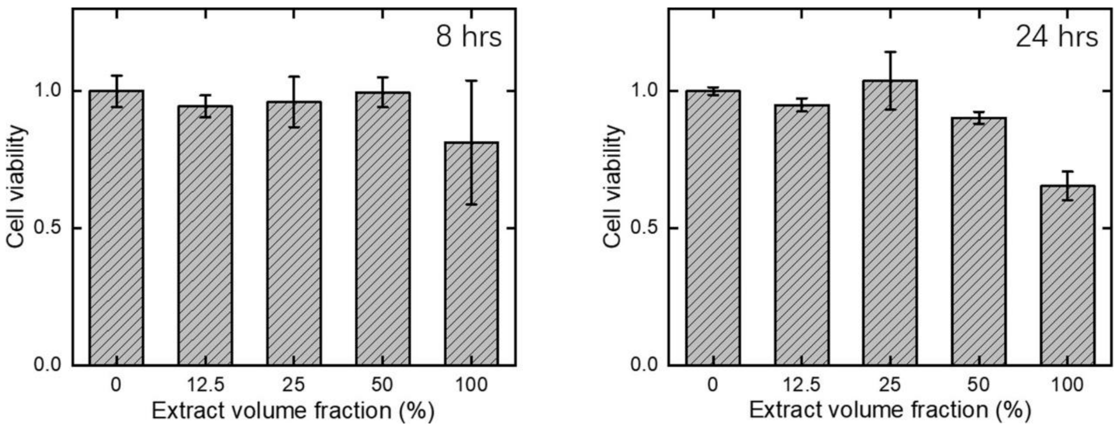


**Supplementary Figure 6.** The CCK8 assay uses a water-soluble tetrazolium salt to quantify the number of live cells by producing an orange formazan dye upon bio-reduction in the presence of an electron carrier. Specifically, an extracting solution of the astral gel is prepared and diluted by DMEM (dulbecco's modified eagle medium) to different extend; fibroblast cells (L929) are then incubated in these mixed solutions for 8 or 24 hours; finally, CCK8 reagents are applied and absorbance at 460 nm recorded to infer cell viability. Our results indicate that the constitute(s) extracted from the astral gel are of certain cytotoxicity that kills ~ 40 % cells in 24 hours. Error bars represent Standard Deviations (n = 3).

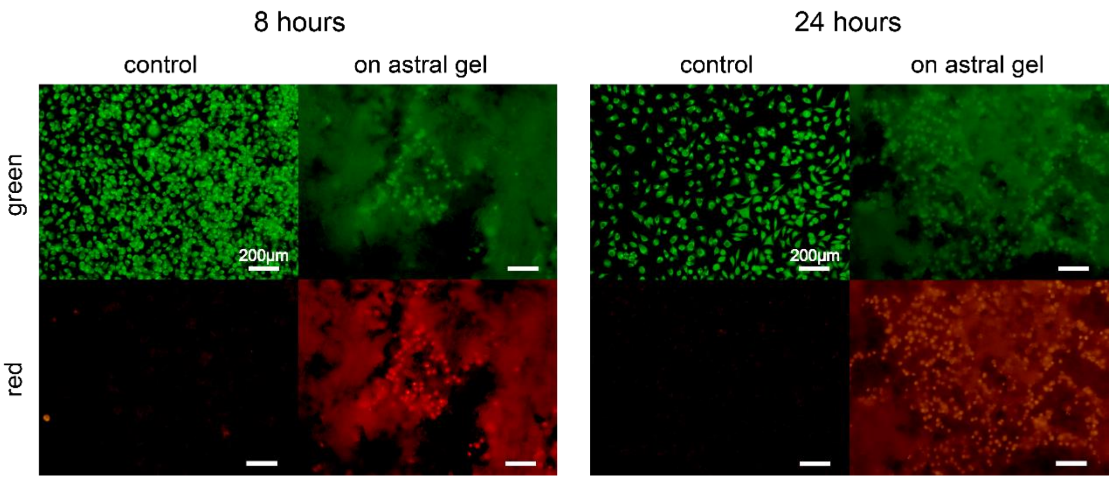


**Supplementary Figure 7.** The live and dead assay employs acridine orange and ethidium bromide to label the live cells as green and the dead as green and red in a fluorescent microscope. L929 cells are seeded on the surface of astral gels (or on the petri dishes as the control) for 8 or 24 hours, after which they are treated with the live/dead reagents and observed. We can see that the cells are alive in the control group but all dead on the astral gels. The fluorescent molecules seem to dye the gel fibers to some extent as well.
