# Supplementary material for: Astral hydrogels mimic tissue mechanics by aster-aster interpenetration
Source: Nat Commun. 2021 Jul 13;12:4277. doi: 10.1038/s41467-021-24663-y (PMC8277779; doi:10.1038/s41467-021-24663-y)
Supplement: Supplementary file 3 — Description of Additional Supplementary Files [file 41467_2021_24663_MOESM3_ESM.docx]

**Description of Additional Supplementary Files**

**Supplementary Movie 1.** Deformation of an aster cluster (ee=0.33) by an optical fiber.

**Supplementary Movie 2.** Deformation of an aster cluster (ee=0) by an optical fiber.

**Supplementary Movie 3.** Deformation of an aster cluster (ee=0.5) by an optical fiber.
